# Supplementary material for: The potential of chemical bonding to design crystallization and vitrification kinetics
Source: Nat Commun. 2021 Aug 17;12:4978. doi: 10.1038/s41467-021-25258-3 (PMC8371141; doi:10.1038/s41467-021-25258-3)
Supplement: Supplementary file 1 — Supplementary Information [file 41467_2021_25258_MOESM1_ESM.pdf]

Supplementary Information

Crystallization and Vitrification Kinetics by Design: The Role of Chemical Bonding

Christoph Persch[1], Maximilian J. Müller[1], Aakash Yadav[1], Julian Pries[1], Natalie Honne[1], Peter Kerres[1], Shuai Wei[1,2], Hajime Tanaka[3,4], Paolo Fantini[5], Enrico Varesi[5], Fabio Pellizzer[6], Matthias Wuttig[1,7,8]

1) I. Institute of Physics, Physics of Novel Materials, RWTH Aachen University, 52056 Aachen, Germany
2) Department of Chemistry, Aarhus University, 8000 Aarhus C, Denmark
3) Institute of Industrial Science, University of Tokyo, 4-6-1 Komaba, Meguro-ku, Tokyo 153-8505, Japan
4) Research Center for Advanced Science and Technology, University of Tokyo, 4-6-1 Komaba, Meguro-ku, Tokyo 153-8505, Japan
5) Micron Technology Inc., Vimercate, Italy
6) Micron Technology Inc., Boise, US
7) Jülich-Aachen Research Alliance (JARA FIT and JARA HPC), RWTH Aachen University, 52056 Aachen, Germany
8) PGI 10 (Green IT), Forschungszentrum Jülich GmbH, 52428 Jülich, Germany

Table of Contents:

I. Table with all data plotted in figures 1 – 4
II. Further information on the chemical bond descriptors
III. Pros and cons to study crystallization from the as-deposited vs. the melt-quenched state
IV. Relationship between the size of the Peierls distortion and the optical properties
V. Optical properties of the amorphous state and its potential relationship to the crystallization speed
VI. Detailed information on the determination of the onset temperature T_o
VII. PTE diagrams and measurement of the minimum time for crystallization

The supplementary information summarizes crucial information about the methods employed to derive the data presented and conclusions drawn. At first, all data displayed in figures 1 – 4 are summarized in one table. Subsequently, further information is provided regarding the chemical bonding descriptors ES and ET and how they were obtained for the solids investigated here.

Furthermore, pros and cons to study crystallization from the as-deposited vs. the melt-quenched state are discussed, followed by a figure which relates the minimum time for crystallization with the reflectance of the amorphous phase. Next, the determination of the onset temperature for the glass transition is depicted in a figure. Finally, the determination of PTE diagrams and the minimum crystallization time are explained, and all PTE diagrams measured are shown.

I. Table with all data plotted in figures 1 – 4

Material (nominal)	Sn/Se (at%)	τ (ns)	T_o (K) 60,000 K/min	T_g (K) 40 K/min	T_m (K)	T_o/T_m (1) 60,000 K/min	ES (1)	ET (1)	R_{cryst} (1)	R_{amorph} (1)
GeTe-GeSe for POT measurements										
GeTe	0	6.14E+02				0.497	1.106	0.190	0.41	0.14
$GeTe_{0.9}Se_{0.1}$	8.3	2.42E+03				0.516	1.126	0.200	0.38	0.14
$GeTe_{0.8}Se_{0.2}$	16.2	9.62E+03				0.534	1.146	0.210	0.42	0.13
$GeTe_{0.7}Se_{0.3}$	25.9	1.14E+04				0.553	1.168	0.222	0.41	0.13
$GeTe_{0.6}Se_{0.4}$	33.4	4.07E+04				0.570	1.188	0.232	0.40	0.13
$GeTe_{0.5}Se_{0.5}$	44.4	1.60E+05				0.595	1.215	0.246	0.37	0.14
$GeTe_{0.4}Se_{0.6}$	51.4	1.27E+06				0.606	1.232	0.254	0.30	0.11
$GeTe_{0.3}Se_{0.7}$	60.5	8.69E+06				0.607	1.255	0.266	0.31	0.11
$GeTe_{0.2}Se_{0.8}$	76.6	*				0.608	1.294	0.286	0.23	0.10
$GeTe_{0.1}Se_{0.9}$	81.9	*				0.605	1.308	0.292		0.14
GeSe	100	*				0.605	1.352	0.315	0.22	0.16
GeTe-GeSe for (F)DSC measurements										
GeTe	0		491**		988	0.497**	1.106	0.190		
$GeTe_{0.875}Se_{0.125}$	13.4		520**		984	0.528**	1.139	0.207		
$GeTe_{0.75}Se_{0.25}$	29.5		543		970	0.560	1.179	0.227		
$GeTe_{0.625}Se_{0.375}$	38.5		556	506	956	0.582	1.201	0.238		
$GeTe_{0.5}Se_{0.5}$	49.5		565	532	933	0.606	1.228	0.252		
$GeTe_{0.375}Se_{0.625}$	74.4		580	549	952	0.609	1.289	0.283		
$GeTe_{0.25}Se_{0.75}$	80.9		584	562	965	0.605	1.305	0.291		
GeSe	100		595	573	986	0.605	1.352	0.315		
GeTe-SnTe for POT measurements										
GeTe	0	6.14E+02				0.497	1.106	0.190	0.41	0.14
$Ge_{0.9}Sn_{0.1}Te$	9.5	3.37E+02				0.490	1.082	0.202	0.47	0.14
$Ge_{0.8}Sn_{0.2}Te$	21.0	2.52E+02				0.483	1.053	0.217	0.46	0.15
$Ge_{0.7}Sn_{0.3}Te$	29.4	1.69E+02				0.480	1.032	0.228	0.46	0.16
$Ge_{0.6}Sn_{0.4}Te$	38.8	8.08E+01					1.009	0.240	0.48	0.19
$Ge_{0.5}Sn_{0.5}Te$	49.6	2.54E+01					0.982	0.255	0.49	0.26
GeTe-SnTe for (F)DCS measurements										
GeTe	0		491**		976	0.497**	1.106	0.190		
$Ge_{0.8}Sn_{0.2}Te$	23.2		471**		982	0.480**	1.048	0.220		
$Ge_{0.7}Sn_{0.3}Te$	32.4		467**		988	0.478**	1.025	0.232		
$GeTe-Sb_2Te_3$										
$Ge_2Sb_2Te_5$		7.89E+01	478**		911	0.525**	1.136	0.172	0.50	0.27
$Ge_3Sb_2Te_6$		1.18E+02	482**		928	0.519**	1.130	0.175	0.44	0.22
$Ge_8Sb_2Te_{11}$		1.32E+02	485**		962	0.504**	1.119	0.182	0.51	0.26

Supplementary Table 1 Collected key data. Overview of the relevant data used for this publication. ES and ET values of the compositions were obtained as explained in the methods section and section II. The crystallization time was measured as explained in detail in section VII. The reflectance of the amorphous and the crystalline phase was measured in the POT using the probe laser. In case crystallization could not be observed in the POT, due to a lack of a discernible reflectance change, measurements of crystallization time were not feasible. These samples are marked with an asterisk in the column for the minimum crystallization time. Measurements of the glass transition temperature were carried out utilizing the FDSC as explained in detail in section VI, values for the melting temperature were taken from published phase diagrams[1]. The T_o/T_m values for POT samples of the GeTe-GeSe and the GeTe-SnTe systems are linear interpolations regarding the closest compositions of the (F)DSC samples. Glass transitions which do not reach into the exothermic region are marked with a double asterisk.

II. Further information on the chemical bond descriptors

The Effective Coordination Number (ECoN) describes a distance-weighted average over all neighbors[2] and is well-suited to characterize trends in the atomic arrangement for systems which undergo local distortions. The value of ECoN for a single atom within a crystal can be calculated according to:

$$ECoN = \sum_j exp\left(1 - \left(\frac{d_j}{d_r}\right)^6\right),$$

where d_j is the distance to the j-th neighbor, and d_r is an effective distance defining the first coordination shell and the sum extending over all neighbors,

$$d_r = \frac{\sum d_j \exp(1-(d_j/d_1)^6)}{\sum \exp(1-(d_j/d_1)^6)}.$$

The ECoN averaged ES can be calculated in an almost identical fashion, where each ES value is weighted by the ECoN contribution corresponding to the respective bonding partner and its distance:

$$\text{ECoN averaged ES} = \frac{\sum_j ES_j \cdot exp\left(1 - \left(\frac{d_j}{d_r}\right)^6\right)}{ECoN}$$

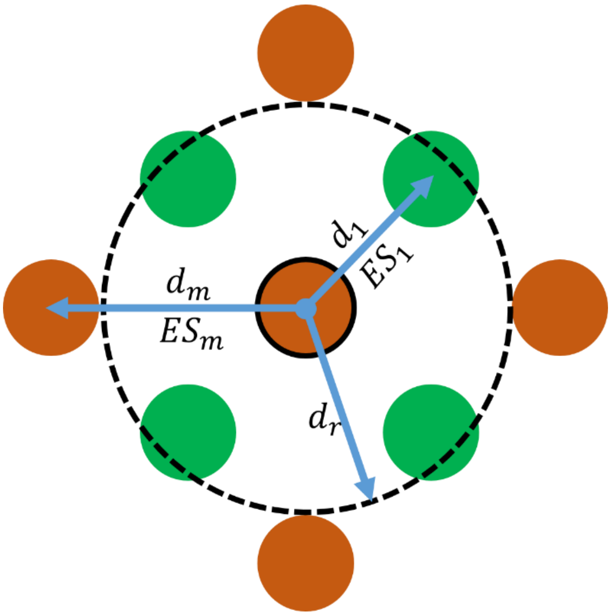

Supplementary Fig. 1 Electrons shared. Sketch describing how to determine the number of electrons shared with neighboring atoms.

III. Pros and cons to study crystallization from the as-deposited vs. the melt-quenched state

The minimum time for crystallization differs between melt-quenched and as-deposited glassy states as has been shown by numerous studies[3–5]. In the last decade a number of reasons have been identified for this difference. Apparently, subcritical nuclei can be frozen in upon melt-quenching[4]. These nuclei

presumably facilitate nucleation and hence speed up the crystallization process. Similar subcritical nuclei and thus a faster crystallization process can be realized upon deposition by other methods like pulsed laser deposition due to the increased kinetic energy of the atoms compared to sputter deposition. At present, the study of the formation of such sub-critical nuclei is beyond the scope of ab-initio molecular dynamics simulations, which employ quenching times which are about 4 orders of magnitude shorter than the experimentally accessible quenching times. Hence, it seems that these computations provide a better model of the as-deposited state of sputtered samples. This is one reason why we focus on the as-deposited state here, since we plan to link the findings presented here to quantum-chemical calculations of the as-deposited amorphous state in the near future.

Furthermore, the characterization of our samples with optical spectroscopy requires samples sizes of several mm, while we can only produce μm-sized regions of the melt-quenched state. Hence, we can only characterize the as-deposited amorphous state with our experimental tools, which are required to relate material properties to crystallization kinetics. Finally, we are presently unable to produce the necessary amounts of melt-quenched material to perform calorimetry measurements, which require about (0.1-2) μg of the material per measurement. Since we rely on laser quenching to produce the melt-quenched phase, only thin layers (up to approximately 50 nm in thickness) can be prepared due to the high absorption of the materials under investigation. Using thicker layers would result in an unsuccessful quenching process since too much heat is absorbed in the top area of the thin film while the rest of the film would hinder heat flow into the substrate, causing the molten area to crystallize rather than staying in the amorphous phase. Due to the limited power of the laser setup, the spot size cannot be increased without losing the ability to melt the sample at all. Thus, producing the amount of melt quenched material required for calorimetric measurements is not feasible. For these three reasons we have studied the crystallization of as-deposited amorphous chalcogenides.

IV. Relationship between the optical properties and the size of the Peierls distortion

Chalcogenides such as GeTe, GeSe or SnTe are characterized by an octahedral-like atomic arrangement where each atom has 6 nearest neighbors, albeit with somewhat different distances. These IV-VI semiconductors have a total of 10 valence electrons, 4s and 6p- electrons. Since the s- and p-electrons hardly hybridize, the bonds are only formed by the 6 p-electrons[6,7]. With 6 neighboring atoms, there is hence only a single electron available to form a bond, a situation very different from ordinary covalent bonding. This also has pronounced consequences for the resulting band structure. With only one electron occupying the corresponding band, the band should be half-filled, i.e. metallic. Yet, there are two mechanisms which create a band gap, electron transfer between the different atoms, as

observed in lead chalcogenides[8], linked to increasing values of ET, or a Peierls distortion as observed in GeTe and GeSe. The Peierls distortion opens a gap, which increases with increasing values of ES. GeSe hence has a significantly larger size of the Peierls distortion than GeTe. This has a pronounced impact on the resulting dielectric function. Both increasing ET (above 0) and increasing ES (above 1) leads to a reduction of the maximum in the absorption, i.e. $\varepsilon_2(\omega)$. This can be explained by a growing dissimilarity of the wave function of the valence and conduction band states. The reduction in the maximum of $\varepsilon_2(\omega)$ reduces the optical reflectance measured.

The same conclusion is also reached by considering the changes of the band gap with increasing ES. Increasing the Peierls distortion (and hence ES) increases the band gap and thus shifts the maximum of $\varepsilon_2(\omega)$ to higher energies. Since the optical sum rule has to be fulfilled, the height of the maximum will decrease with increasing amplitude of the distortion, reducing the optical reflectance.

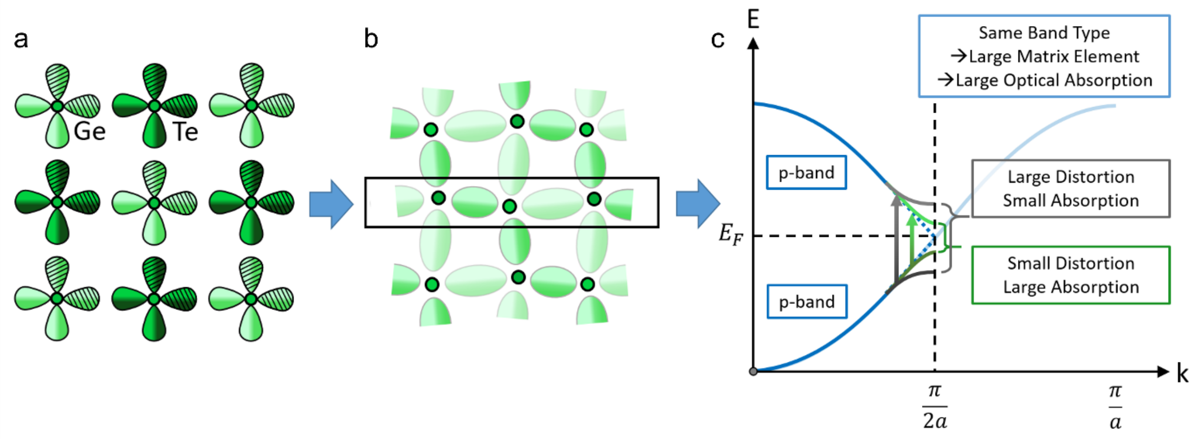

Supplementary Fig. 2 Bond formation and resulting band structure in GeTe. a Atomic orbits of Ge and Te responsible for bond formation in the solid are depicted. **b** The atomic arrangement in (001) plane is shown, where the formation of σ-bonds between adjacent atoms are responsible for bond formation. **c** The σ-bonds are occupied by about an electron (ES ≈ 1), resulting in a metallic band (blue curves on the right side). However, the Peierls distortion results in a small bandgap. The figure is adapted from[8].

V. Optical properties of the amorphous state and its potential relationship to the crystallization speed

As can be seen in Supplementary Fig. 3, there are significant differences in the reflectance of the different amorphous samples, especially for the GST based compounds and the GeTe-SnTe alloys, which lead to very similar crystallization times. On the other side, there are modest changes in reflectance of the amorphous GeTe-GeSe compounds, which lead to striking differences in minimum crystallization time. We can hence conclude that the minimum crystallization time is not closely related

to the reflectance of the amorphous samples, but instead is closely related to the reflectance of the crystalline samples.

This aspect has recently been investigated in more detail for a series of samples along the pseudo-binary line between GeTe and GeSe, as well as two other similar lines[9]. This study has shown unequivocally that the optical properties hardly change upon the transition from amorphous GeTe to amorphous GeSe. Instead, a much more pronounced change of optical properties has been observed for the crystalline samples along the GeTe-GeSe pseudo-binary line. Increasing the Ge content leads to a monotonous decrease of the optical dielectric constant ε_∞, which can be attributed to an increase of the Peierls distortion in the crystalline phase. At a critical Se concentration of around 60%, two different crystalline phases are observed which differ significantly in optical reflectivity and optical dielectric constant ε_∞. These two different samples also differ in their degree of Peierls distortion[9]. This indicates that there are much more pronounced changes of the bonding and structure and hence also physical properties along the crystalline Ge-Te-GeSe pseudo-binary line than along the amorphous GeTe-GeSe pseudo-binary line. Apparently, the changes in optical properties of the crystalline samples are thus linked to changes in crystallization kinetics. While this conclusion appears reasonable, further data such as quantum-chemical calculations to characterize the bonding in amorphous phase change materials are needed to quantify trends for the bonding in this phase with stoichiometry as well.

In this context, the finding reported in Fig. 3b also raises questions. There it was shown that amorphous GST alloys are characterized by an increased onset temperature T_{ro} for glass formation. One can wonder if this is due to an increased Peierls distortion in amorphous GST alloys caused by the additional disorder in ternary compounds. This increased disorder should facilitate larger Peierls distortions and thus an increased stability of the amorphous phase against crystallization. Yet, at elevated temperatures we expect a fragile to strong transition of the viscosity. Yet, to validate this speculation a more detailed analysis of the atomic arrangement and the dielectric function, as well as the temperature dependence of the viscosity would be required to turn this plausible hypothesis into a proven fact.

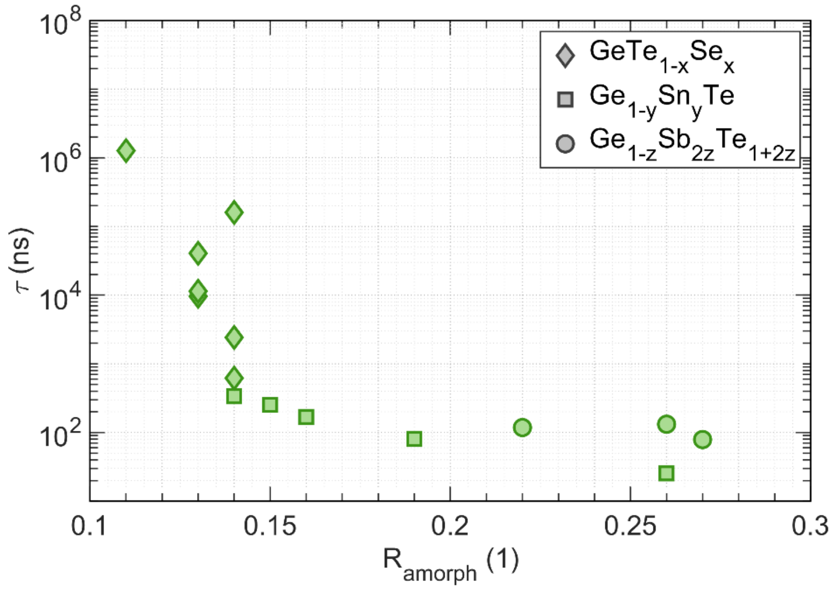

Supplementary Fig. 3 Minimum time for crystallization τ versus the reflectance of the amorphous as-deposited phase.

VI. Detailed information on the determination of the onset temperature T_o

Differential scanning calorimetric measurements were performed with a Mettler Toledo Flash DSC 1 (FDSC). The amorphous material was heated at a constant heating rate of 60,000 K/min from 30 °C to 450°C. With the FDSC the power required to assure a constant heating rate is measured. The same heating procedure is repeated with the now crystallized sample and subtracted from the initial scan to obtain the excess specific heat capacity as a function of temperature. All measurements were performed on single flat flakes of amorphous as-deposited material. From the so obtained thermograms, specific features like the glass transition (if not obscured by crystallization) and crystallization can be identified, see Supplementary Fig. 4. For all as-deposited materials measured here, an exothermic enthalpy relaxation was observed, causing the DSC signal to depart from the zero line into the negative regime (endothermic is up). The enthalpy relaxation reaches a minimum or a plateau before the beginning glass transition causes an upward trend. Upon ongoing glass transition, the signal would become endothermic and would show an endothermic overshoot before entering the undercooled liquid (UCL). The overshoot is observed for GeSe-rich compounds while the plateau of the UCL in the excess heat capacity (or power) is already obscured by crystallization. By increasing the GeTe content, less of the glass transition is visible until the signal does not reach the endothermic regime anymore and the glass transition is obscured by crystallization, see Supplementary Fig. 4. No endothermic signal and thus no glass transition is observed for GeTe-rich compounds of the GeTe:GeSe system and the material systems GeTe:SnTe and GeTe:Sb_2Te_3. However, the deviation from the enthalpy relaxation minimum occurs at the very beginning of the glass transition, so obtaining the

onset temperature of this deviation T_o yields a temperature of the first sign of the glass transition as indicated in Supplementary Fig. 4. Therefore, this onset temperature T_o is related to the glass transition T_g at that heating rate of 60,000 K/min and should show a similar dependence on stoichiometry. For each material, the onset temperature T_o is averaged over ten measurements.

In conventional DSC (Diamond DSC, PerkinElmer) the same heating and measuring procedure was applied to the same compounds as in FDSC measurements, employing a heating rate of 40 K/min. The apparent glass transition temperature T_g of the as-deposited material was measured by an onset construction. Please note that this apparent glass transition temperature is not the standard glass transition temperature as it is measured from the as-deposited amorphous phase and not from a standard glass. In the conventional DSC measurements, the glass transition was either visible and the signal became endothermic prior to crystallization, or crystallization was observed before the enthalpy relaxation reached its minimum so that an onset temperature similar to the T_o obtained from FDSC measurements described above was not obtained.

The reduced onset temperature T_{rg} shows the same trend as the reduced glass transition temperature T_{rg} as Supplementary Fig. 5 demonstrates. Hence, the stoichiometry dependence of the reduced glass transition temperature can be derived from the stoichiometry dependence of the reduced onset temperature.

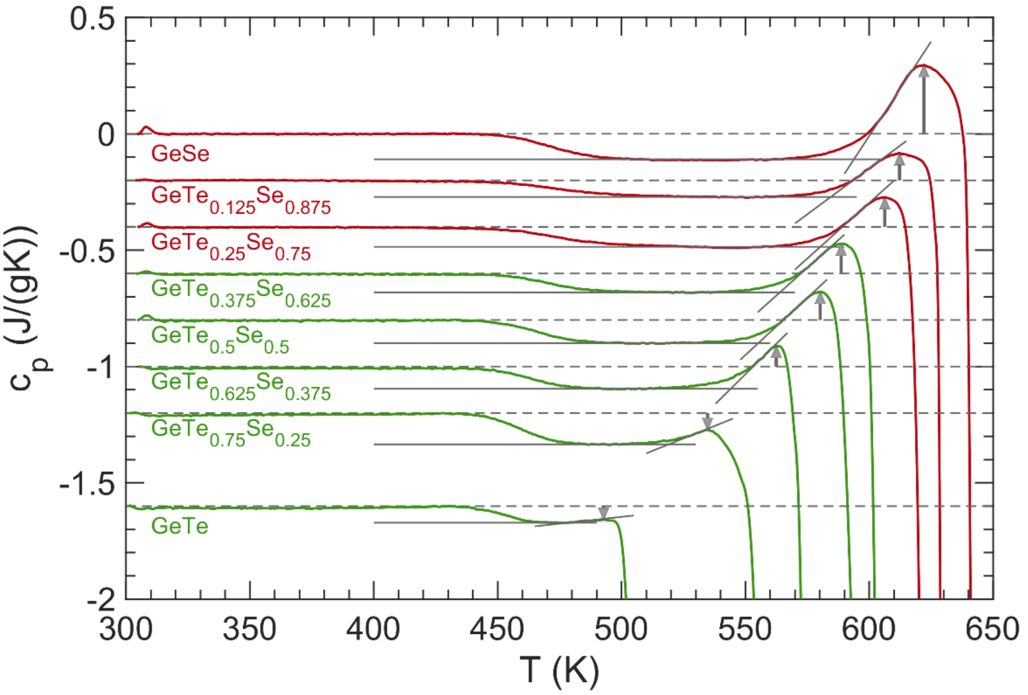

Supplementary Fig. 4 Excess specific heat. Temperature dependence of the excess specific heat capacity at constant pressure measured at a heating rate of θ = 60,000 K/min measured by FDSC. The measurement of a crystalline reference is subtracted in each trace. The temperature is calibrated to the onset temperature of melting for an Indium flake at the same heating rate used for measurements. The maximum endothermal signal of the glass transition is indicated by an arrow. For Se-rich samples the glass transition is well discernible. The compositions shown are nominal. For exact compositions refer to Supplementary Table 1, please.

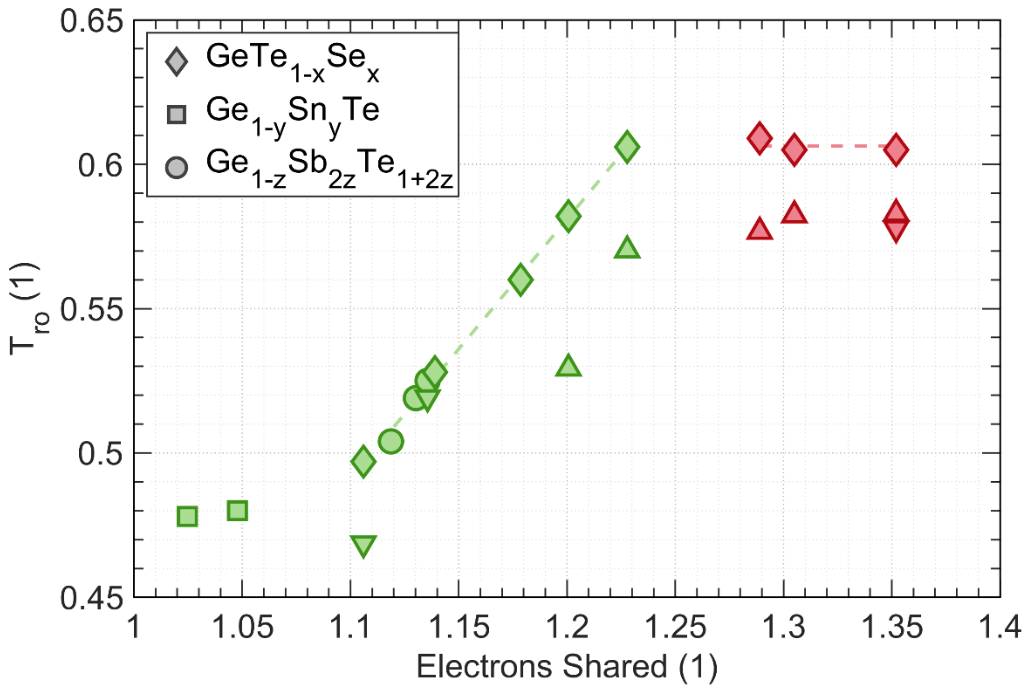

Supplementary Fig. 5 Glass transition combined with literature data. Dependence of the reduced glass transition T_{rg} (triangles) and the reduced onset temperature T_{ro} (all other symbols) for glass formation upon ES (for the crystalline phase). T_{ro} was measured at a heating rate of 60,000 K/min, while T_{rg} is measured at 20 K/min (triangles pointing up: present data, pointing down: literature data). Replacing Te in GeTe by Se leads to a significant increase in glass-forming ability as characterized by T_{ro} and T_{rg}.

VII. PTE diagrams and measurement of the minimum time for crystallization

The minimum time for crystallization τ has been determined with the phase change optical tester (POT), a pump-probe laser setup that allows for a continuous measurement of sample reflectance, while the sample is exposed to pump laser pulses. When a rectangular laser pulse hits the sample, the absorbed energy results in local heating of the illuminated area. Depending on the applied pulse power and pulse length, the sample can be switched between its amorphous and its crystalline phase. The change in the dielectric function upon switching results in a change of sample reflectance measured by the probe laser before and after the pump pulse. To prevent evaporation of the material during heating, the material under investigation is incorporated into a layer stack comprised of two additional buffer layers made of $(ZnS)_{80}(SiO_2)_{20}$. While the top layer contains the material upon heating, the bottom layer increases the thermal resistance, thus limiting the heat flux from the material under investigation into the silicon substrate.

Measuring the reflectance of the sample before (R_{pre}) and after (R_{post}) the application of a heating pulse yields the contrast (C):

$$C = \frac{R_{post} - R_{pre}}{R_{pre}} \cdot 100 \, .$$

Plotting the contrast in a 2D-map using the applied pulse length and pulse power for the x- and y-axis yields a power-time-effect diagram (PTE-diagram). Since an increased pulse power is correlated with an increased temperature in the sample, the y-axis reflects the temperature within the material. Thus, the pulse length is correlated with the evolution of the material with time. The effective central power density of the laser E is given by:

$$E = \frac{8}{\pi} \cdot \frac{P}{d},$$

Where d is the diameter ($1/e^2$) of the laser beam of 2.3 µm.

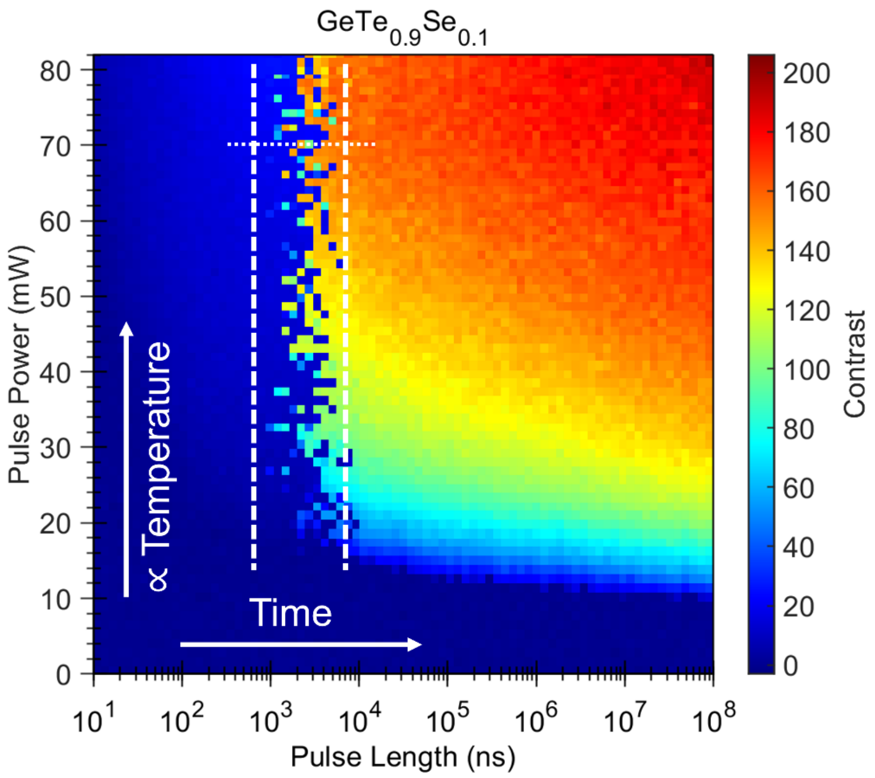

Supplementary Fig. 6 PTE-diagram of the as-deposited amorphous $GeTe_{0.9}Se_{0.1}$ sample measured with the POT. Positive contrast values correspond to crystallization due to the heating pulse, an increased contrast correlates with an increased amount of crystallized material. The dashed white lines indicate the pulse-length regime where crystallization sets in. The dotted white line indicates the subset of pulse length used to precisely measure the minimum time for crystallization.

In Supplementary Fig. 6, an exemplary PTE-diagram is shown. Here, an as-deposited amorphous sample has been used, pump pulses of varying length and power have been applied and the reflectance before and after each pulse has been analyzed to obtain the PTE-diagram. For each measurement point, the sample has been moved to illuminate a new region of the sample. PTE-diagrams of each

material investigated are shown in Supplementary Fig. 8 and 9. From the PTE diagrams it is evident, that crystallization (as indicated by a positive contrast) does not set in immediately, but only after a certain time has passed. Remarkably, the onset of crystallization is rather constant with respect to pulse power above a certain threshold. As shown in Supplementary Fig. 6, the onset of crystallization involves some stochasticity, i.e. pulse to pulse variation. Consequently, defining a minimum time τ it takes the sample to form crystallites should also consider the stochasticity of crystallization. To obtain τ, the onset of crystallization is investigated in detail. First, based on the PTE-diagrams, suitable pump-parameters (pulse length and pulse power) have been selected to determine τ from repeated measurements of the change in sample reflectance upon local heating. Using these parameters, the pulse length interval has been divided into 40 equidistant (on a logarithmic scale) segments and the contrast has been measured 40 times for each segment. For each measurement, the sample has been moved to a new position, so a total of 1,600 positions has been probed. From these experiments two reflectance levels can be identified, the lower level, where no crystallites have formed (R_{min}), whereas the upper level corresponds to measurements, where the crystallites completely cover the illuminated area (R_{max}). With these two levels, R_{min} and R_{max}, a threshold value R_{thr} is calculated as follows:

$$R_{thr} = 0.1 \cdot (R_{max} - R_{min})$$

Contrast values above R_{thr} are counted as crystallization events, whereas contrast values below R_{thr} are not. Consequently, the probability of crystallization P_{cryst} can be deduced:

$$P_{cryst}(t) = 100 \cdot \frac{N_{cryst}(t)}{N_{tot}},$$

where N_{tot} and N_{cryst} are the total number of measurements at a given pulse length t and the number of contrast values above R_{thr}, respectively. The result of plotting the probability of crystallization versus the applied pulse length t for $GeSn_{0.5}Te_{0.5}$ at a laser power of 70 mW is presented in Supplementary Fig. 7.

From the probability of nucleation, the minimum time for crystallization is deduced by fitting a Gompertz function to the data and calculating the pulse length where crystallization is observed with a probability of 50%.

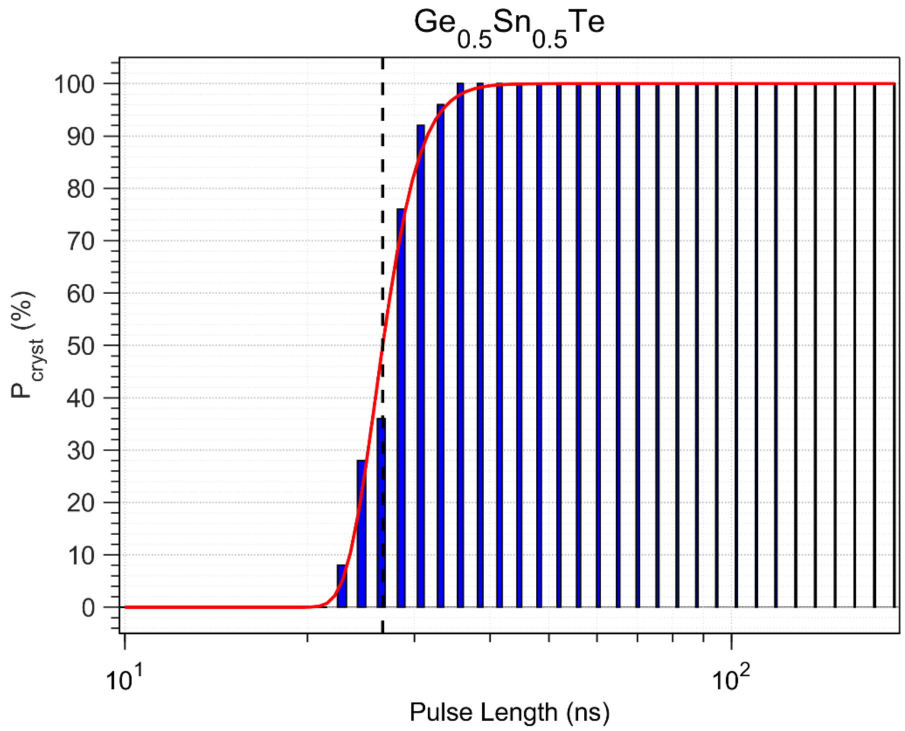

Supplementary Fig. 7 Probability of crystallization plotted versus the applied pulse length at a laser power of 70 mW for $GeSn_{0.5}Te_{0.5}$. The red solid line is a fit to the data (Gompertz function), the black dashed line indicates the pulse length where a probability of crystallization of 50% is observed, termed minimum time for crystallization τ.

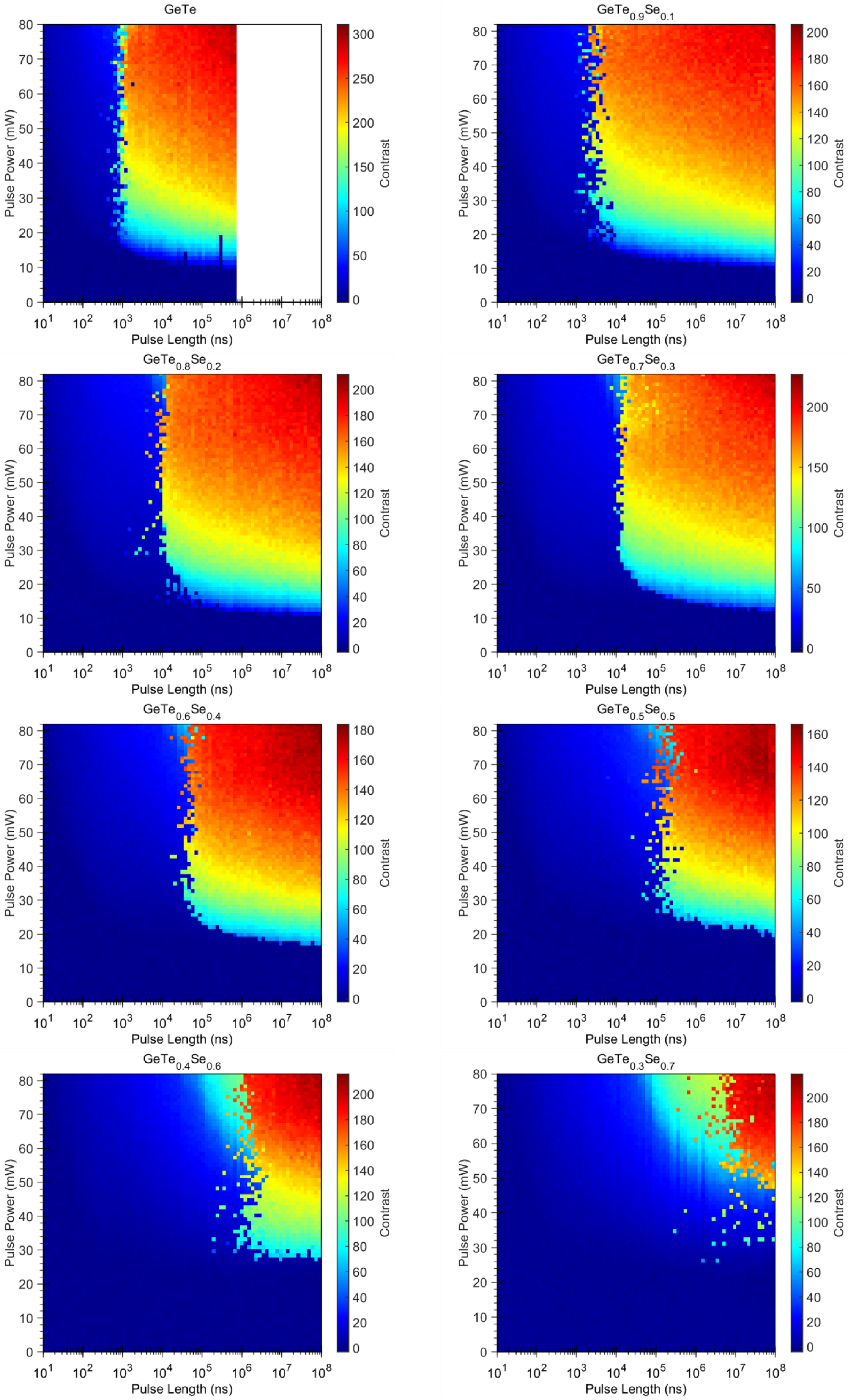

GeTe
GeTe0.9Se0.1
GeTe0.8Se0.2
GeTe0.7Se0.3
GeTe0.6Se0.4
GeTe0.5Se0.5
GeTe0.4Se0.6
GeTe0.3Se0.7
Pulse Power (mW)
Pulse Length (ns)
Contrast

Supplementary Fig. 8 PTE-diagrams of the $GeTe_{1-x}Se_x$ material system. The contrast is calculated from the reflectance before and after the application of a heating pulse.

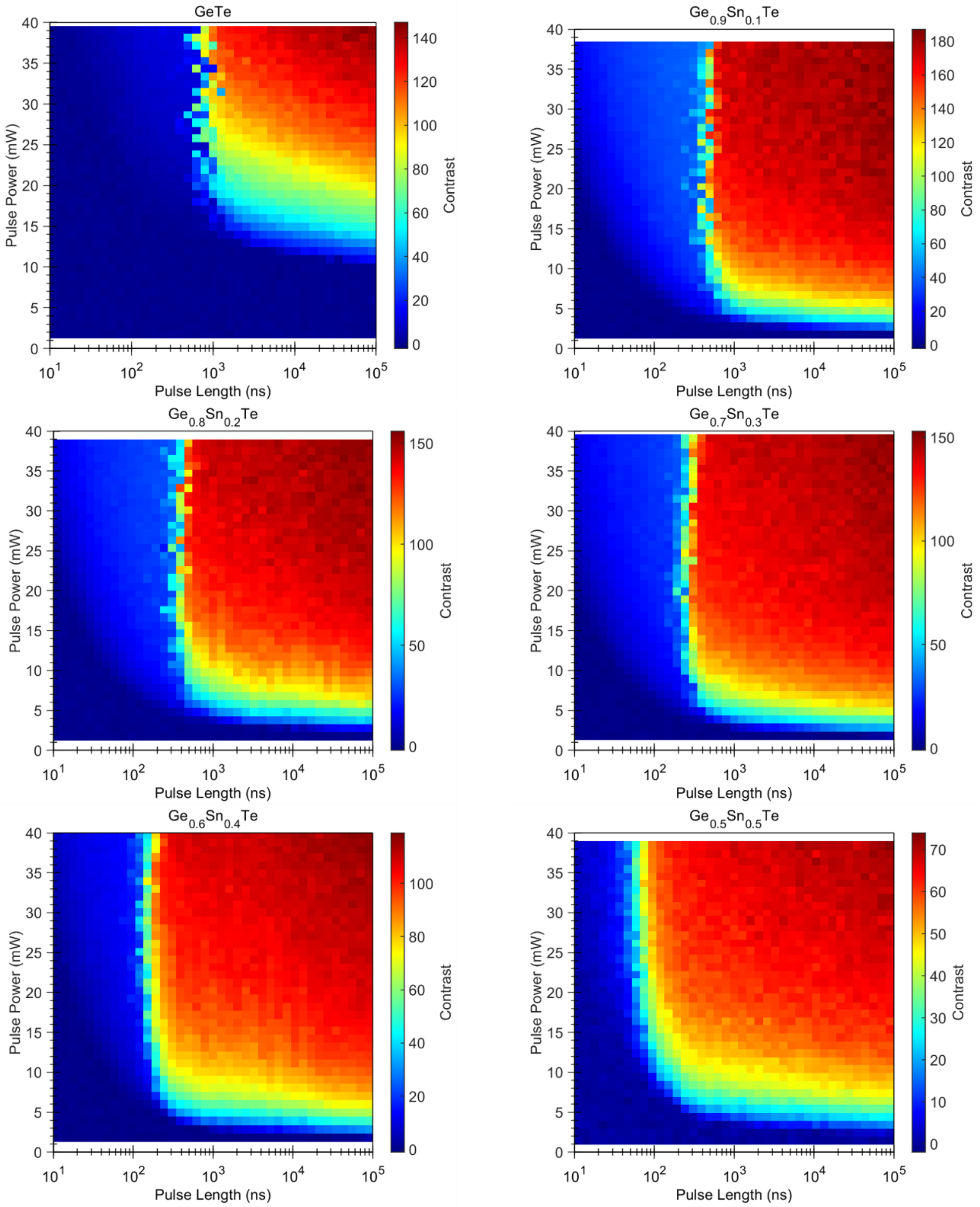

Supplementary Fig. 9 PTE-diagrams of the $Ge_{1-x}Sn_xTe$ material system. The contrast is calculated from the sample reflectance before and after the application of a heating pulse.

1. Marsh, K.N., Frenkel, M., Wilhoit, R.C., & Zhang, Z. *Landolt-Börnstein: Numerical Data and Functional Relationships in Science and Technology - New Series (8A)*.

2. Hoppe, R. Effective coordination numbers (ECoN) and mean fictive ionic radii (MEFIR). *Zeitschrift für Kristallographie - Crystalline Materials* **150**, 23–52 (1979).

3. Salinga, M. et al. Measurement of crystal growth velocity in a melt-quenched phase-change material. *Nature Communications* **4**, 2371 (2013).

4. Lee, B.-S. et al. Observation of the Role of Subcritical Nuclei in Crystallization of a Glassy Solid. *Science* **326**, 980–984 (2009).

5. Wei, J. & Gan, F. Theoretical explanation of different crystallization processes between as-deposited and melt-quenched amorphous Ge2Sb2Te5 thin films. *Thin Solid Films* **441**, 292–297 (2003).

6. Kooi, B. J. & Wuttig, M. Chalcogenides by Design: Functionality through Metavalent Bonding and Confinement. *Advanced Materials* **32**, 1908302 (2020).

7. Lencer, D., Salinga, M. & Wuttig, M. Design Rules for Phase-Change Materials in Data Storage Applications. *Advanced Materials* **23**, 2030–2058 (2011).

8. Guarneri, L. et al. Metavalent Bonding in Crystalline Solids: How Does It Collapse?. *Adv. Mater.* 2102356, https://doi.org/10.1002/adma.202102356 (2021).

9. Guarneri, L. et al. Metavalent bonding in crystalline solids: how does it collapse? *arXiv:2008.10219 [cond-mat]* (2020).
